# Supplementary material for: MetaRibo-Seq measures translation in microbiomes
Source: Nat Commun. 2020 Jun 29;11:3268. doi: 10.1038/s41467-020-17081-z (PMC7324362; doi:10.1038/s41467-020-17081-z)
Supplement: Supplementary file 10 — Supplementary Data 7 [file 41467_2020_17081_MOESM10_ESM.zip › File2/Confidence_High_Taxonomy/180233_out.krona.html]

Javascript must be enabled to view this page.

members
magnitude
magnitudeUnassigned
count
unassigned
taxon
rank

180233\_out

9

9
superkingdom
2

phylum
9
1239

186801
class
9

order
9
186802

31979
9
family

1485
9
genus

411489

SRS019161\_contig\_number\_32607SRS053356\_contig\_number\_43735SRS077231\_contig\_number\_14818SRS078419\_contig\_number\_contig-100\_11007.11007SRS147346\_contig\_number\_62327
species
5

2292996

SRS013951\_contig\_number\_8908SRS015217\_contig\_number\_contig-100\_433.185663SRS024132\_contig\_number\_contig-100\_194.220758SRS045713\_contig\_number\_contig-100\_2493.65902
species
4
